# A rapid magnetic bead-based immunoassay for sensitive determination of diclofenac

**DOI:** 10.1007/s00216-021-03778-7

**Published:** 2021-11-20

**Authors:** Alexander Ecke, Tanja Westphalen, Jane Hornung, Michael Voetz, Rudolf J. Schneider

**Affiliations:** 1 Department of Analytical Chemistry; Reference Materials, Bundesanstalt für Materialforschung und -prüfung (BAM), 12489 Berlin, Germany; 2 Department of Chemistry, Humboldt-Universität zu Berlin, 12489 Berlin, Germany; 3 sifin diagnostics gmbh, 13088 Berlin, Germany; 4 Technische Universität Berlin, Faculty III Process Sciences, 10623 Berlin, Germany

**Keywords:** Immunoassay, Magnetic beads, Diclofenac, Water analysis, LC–MS/MS

## Abstract

Increasing contamination of environmental waters with pharmaceuticals represents an emerging threat for the drinking water quality and safety. In this regard, fast and reliable analytical methods are required to allow quick countermeasures in case of contamination. Here, we report the development of a magnetic bead-based immunoassay (MBBA) for the fast and cost-effective determination of the analgesic diclofenac (DCF) in water samples, based on diclofenac-coupled magnetic beads and a robust monoclonal anti-DCF antibody. A novel synthetic strategy for preparation of the beads resulted in an assay that enabled for the determination of diclofenac with a significantly lower limit of detection (400 ng/L) than the respective enzyme-linked immunosorbent assay (ELISA). With shorter incubation times and only one manual washing step required, the assay demands for remarkably shorter time to result (< 45 min) and less equipment than ELISA. Evaluation of assay precision and accuracy with a series of spiked water samples yielded results with low to moderate intra- and inter-assay variations and in good agreement with LC–MS/MS reference analysis. The assay principle can be transferred to other, e.g., microfluidic, formats, as well as applied to other analytes and may replace ELISA as the standard immunochemical method.

**Graphical abstract:**

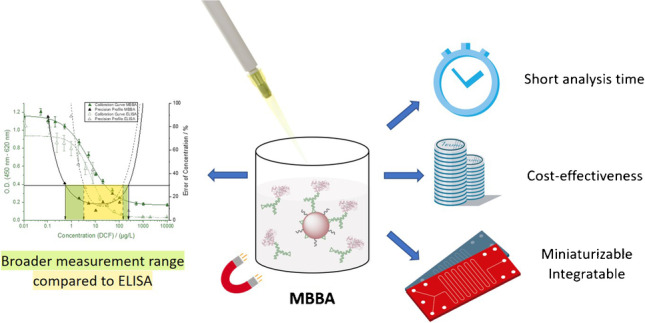

**Supplementary Information:**

The online version contains supplementary material available at 10.1007/s00216-021-03778-7.

## Introduction

**Fig. 1:**
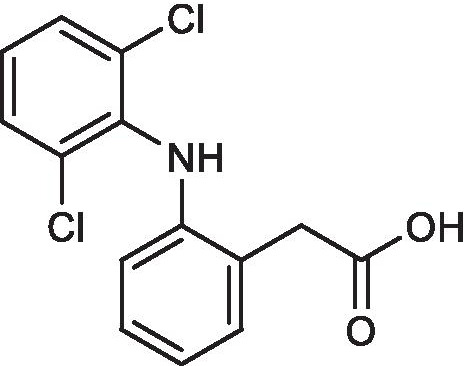
Chemical structure of diclofenac (DCF)

The nonsteroidal anti-inflammatory drug (NSAID) diclofenac (DCF, Fig. 1) has been used frequently in the treatment of rheumatic diseases, inflammations, and fever, as well as acute and chronic pain since its introduction in the 1970s [1, 2]. Together with ibuprofen and naproxen, DCF is among the most commonly sold non-aspirin NSAIDs in several countries worldwide over the past years [3, 4]. Accordingly, the discharge of DCF via wastewater is relatively high considering that the majority of orally administered DCF is excreted unaltered or metabolized via urine (65–70%) or feces (20–30%) and DCF applied cutaneously is mainly washed away (> 90%) [5, 6].

Insufficient degradation in wastewater treatment plants (WWTPs) promotes introduction of DCF into surface waters where its ecotoxicological effects can lead to disruption of whole biosystems [6–12]. As a consequence, DCF has been proposed as a priority substance and was added to the EU watchlist for substances of concern requiring Union-wide monitoring in the field of water policy [13]. In this context, environmental quality standards for DCF in inland waters of 100 ng/L and 10 ng/L in all other surface waters were proposed [14]. The concentration values of DCF found in various surface waters, however, exceed these limits with values in the low µg/L range reported for rivers in Europe, Asia, Africa, America, and even Antarctica (for a review, see [12]). As a result, DCF can also reach water bodies that are used for drinking water preparation which is highly alarming in concern of human health. So far, concentrations in the two- to three-figure ng/L-range are already found in many groundwater and drinking water samples [12]. Strategies for more effective degradation of DCF in WWTPs to prevent introduction into environmental water, and thus into drinking water, have been reported before [15–20], but are not yet applied comprehensively so that fast and reliable analysis methods combined with local countermeasures in case of contamination are still required.

The method of choice for the determination of DCF in water samples with high accuracy is LC–MS/MS in combination with solid-phase extraction for sample preparation [13, 21]. However, these methods are time-consuming and expensive, and require specially trained personnel. In this regard, immunoassays based on the binding of the respective analyte to an analyte-selective antibody and the quantification of these binding events have gained importance as they enable fast (high-throughput) and cost-effective (low amounts of reagents required) analyses. Moreover, thanks to portable detection units, measurements can also be performed outside of the lab at the point of care.

First and foremost, a specific antibody is needed for effective immunochemical detection. Several anti-DCF antibodies with different affinities and sensitivities have been developed in recent decades [22–24]. Based on this, immunoassays were set up in the well-investigated and validated ELISA format to investigate binding conditions and cross-reactivity [22, 23, 25]. For on-site application, though, the conventional microplate-based ELISA is still too lengthy and dependent on bulky equipment like shakers, washers, and microplate readers. This is why efforts have been made to transfer the immunochemical detection to faster and/or more mobile platforms. Faster immunoassays developed for DCF comprise an upconversion-linked immunosorbent assay (ULISA) [26], a fluorescence polarization immunoassay (FPIA) [27], and a suspension array fluorescence immunoassay (SAFIA) [28]. Steps forward to mobile and automated sensing of diclofenac have been made with several immunosensors for the detection of DCF reported in recent years [29–35].

In this context, the application of magnetic particles appears promising as these can be immobilized and released easily by applying an external magnetic field. By coupling one component of the immunoassay to the particle surface, the assay could be implemented into an immunosensor for automated online detection as shown for other analytes previously [36–38]. Usually, the antibody is immobilized on the bead surface (direct approach) to set up a magnetic bead-based assay which is often more straightforward [39–42]. For some analytes, however, coupling of the analyte molecule to the particle surface (indirect approach) is more feasible [43–45]. For DCF, it could be mandatory. To date, no direct immunoassay for DCF has been reported. Concerning peroxidase-based immunoassays, the reason for this may lie in DCF’s property of serving as a substrate for peroxidase [15]. Because a direct immunoassay for DCF would require an enzyme tracer, i.e., a conjugate of DCF coupled to peroxidase, this may be the main obstacle as the effects of coupling an enzyme to its substrate are not yet studied in detail.

This is why we chose an indirect approach to set up an MBBA for the quantification of DCF in water samples with the robust anti-DCF antibody F01G21 [24]. A novel synthetic strategy for DCF coupling to the bead surface was used to tune binding properties of the antibody in order to allow for a more sensitive detection. In combination with the advantages of magnetic particles, a fast and reliable immunoassay with potential for further application, e.g., implementation into an immunosensor, is presented.

## Materials and methods

### General equipment

Ultrapure water was taken from a Merck Millipore (Darmstadt, Germany) Milli-Q Reference water purification system. Weighing was performed on a Sartorius (Göttingen, Germany) Research R180D-*D1 or Cubis® Advanced MCA225S-2S00-I analytical balance. Adjustment of pH values was done with a SevenEasy pH meter S20 from Mettler Toledo (Columbus, OH, USA).

### Preparation of DCF-coupled beads

Bead preparation and all related reactions were performed in 2-mL centrifuge tubes from Eppendorf (Hamburg, Germany). Shaking of reaction mixtures was executed in a ThermoMixer C from Eppendorf, and centrifugation was carried out using an Eppendorf Centrifuge 5417R. For washing, a BioMag® Multi-6 Microcentrifuge Tube Separator from Polysciences (Hirschberg an der Bergstraße, Germany) was used.

#### NHS-activation of DCF

As reported before [28], diclofenac sodium salt (Sigma-Aldrich, Steinheim, Germany) was dissolved in dry *N,N*-dimethylformamide (DMF, Sigma-Aldrich) under argon atmosphere to a final concentration of 1/6 mol/L. Stock solutions of *N*-hydroxysuccinimide (NHS, Merck) and *N,N’*-dicyclohexylcarbodiimide (DCC, Sigma-Aldrich) of 1/2 mol/L each in DMF were prepared under argon. To the DCF solution, NHS solution (1.2 eq), a spatula tip of *N,N’*-disuccinimidyl carbonate (DSC, Sigma-Aldrich), and DCC solution (1.2 eq) were added in this particular order under argon. The resulting solution was shaken in the dark at RT and 750 rpm for 18 h.

Afterwards, the mixture was centrifuged at 4 °C and 4000 rpm for 10 min in order to separate the precipitated dicyclohexylurea. The supernatant solution was used directly for coupling.

#### Coupling to magnetic beads

A suspension of amino-functionalized magnetic microparticles (Sigma-Aldrich, 100 µL) in 500 µL absolute ethanol (Th. Geyer, Renningen, Germany) and 500 µL 0.1 M sodium bicarbonate (Sigma-Aldrich) was prepared. Consecutively, 100 µL of a solution of 0.5 mol/L glutaric anhydride (Merck) in absolute ethanol and 5 µL of the above described DCF active ester solution were added. The resulting mixture was shaken at RT and 900 rpm for 20 h.

Thereafter, beads were washed once with Milli-Q water (1 mL) and thrice with absolute ethanol (1 mL) using a magnetic separator to hold the beads while removing the supernatant. Beads were then resuspended in absolute ethanol (1 mL) and stored at 4 °C until further use.

### Buffers

All buffers were prepared in Milli-Q water and stored in amber glass bottles at room temperature (RT, 22 ± 1 °C) unless stated otherwise. The pH values were adjusted using 6 M hydrochloric acid (Merck) or 5 M sodium hydroxide solution (J.T.Baker, Phillipsburg, NJ, USA).

- Phosphate-buffered saline (PBS), pH 7.6: 10 mM sodium phosphate monobasic dihydrate (Sigma-Aldrich), 70 mM sodium phosphate dibasic dihydrate (Sigma-Aldrich), 145 mM sodium chloride (Sigma-Aldrich).
- Washing buffer, pH 7.6: 0.75 mM potassium phosphate monobasic (Sigma-Aldrich), 6.25 mM potassium phosphate dibasic (Sigma-Aldrich), 0.025 mM potassium sorbate (Sigma-Aldrich), 0.05% Tween 20 (Serva, Heidelberg, Germany).
- Assay buffer (Tris–EDTA), pH 7.6, storage at 4 °C: 125 mM tris(hydroxymethyl)-aminomethane (Tris, Merck), 187.5 mM sodium chloride, 13.375 mM ethylenediaminetetraacetic acid disodium salt dihydrate (Na_2_EDTA·2H_2_O, Sigma-Aldrich).
- Citrate buffer, pH 4.0, storage at 4 °C: 220 mM sodium citrate monobasic (Sigma-Aldrich).
- TMB stock solution in dry *N,N*-dimethylacetamide (DMA, Sigma-Aldrich), storage under argon at 4 °C: 8 mM tetrabutylammonium borohydride (Sigma-Aldrich), 40 mM 3,3’,5,5’-tetramethylbenzidine (TMB, Serva).

### Immunoreagents

Polyclonal sheep anti-mouse IgG (H + L chain) antibody with horseradish peroxidase (HRP) label (secondary antibody, R1256HRP) was obtained from OriGene Technologies (Rockville, MD, USA). Mouse anti-DCF antibodies (isotype IgG1) F01G21 and SK60-2E4 were produced by fusion of mouse myeloma cells (AG8 [24] or SP2/0-AG14, respectively) and splenocytes from BALB/c mice, both obtained from the University of Salzburg [24].

The antibody F01G21 was further labeled with HRP using the periodate method as described by Wilson and Nakane [46]. Required chemicals sodium carbonate, sodium bicarbonate, and ammonium sulfate were from Carl Roth (Karlsruhe, Germany); sodium periodate and sodium cyanoborohydride from Sigma-Aldrich, ethylene glycol from Serva, and HRP from Roche (Basel, Switzerland). The antibody solution was concentrated to about 5 mg/mL in an Amicon® Stirred Cell Model 8010 (10 mL) equipped with an Ultrafiltration Disc (30 kDa) from Merck Millipore (Burlington, MA, USA). The labeling reaction was carried out in a glass vial equipped with a magnetic stirrer. The labeled antibody was purified in PBS using PD-10 or PD MiniTrap desalting columns with Sephadex G-25 resins from GE Healthcare (Chicago, IL, USA). Antibody concentration (2.79 mg/mL) and coupling ratio (2.65) were determined by UV/Vis absorption measurements in UV cuvettes micro from Brand (Wertheim, Germany) with a BioMate 3 UV–Vis Spectrophotometer from Thermo Fisher Scientific (Waltham, MA, USA). The product was stabilized with 0.04% thiomersal (Serva) and 2% fetal bovine serum (Life Technologies, Carlsbad, CA, USA), and stored in aliquots at 4 °C for up to 1 month or at –20 °C for long-term storage.

### Standards and samples

Firstly, a stock solution of DCF-Na analytical standard (Sigma-Aldrich) in absolute ethanol with a mass concentration of approx. 1 g/L was prepared gravimetrically by weighing both solid and the solvent. From this solution, serial dilutions in Milli-Q water were made volumetrically to prepare DCF standards in the concentration range from 10 mg/L to 1 ng/L.

Spiked water samples were prepared by pre-diluting the stock solution of DCF-Na analytical standard in Milli-Q water and successive dilution in the respective sample to the desired concentration. Water samples were taken as specified in Table 1.

**Table 1:** Details on water samples and sampling procedure

Sample	Sampling day	Sample description	Sampling site
Pure water	08.06.2021	From water purification system	BAM building 8.05, room 395C
Drinking water	08.06.2021	From water cooler	BAM building 8.05
Mineral water	09.06.2021	Bottled water, nonsparkling, Lichtenauer Pur, BBD: 31.08.21	-
Tap water	08.06.2021	From laboratory water tap	BAM building 8.05, room 394
Groundwater	07.06.2021	From water well with an electric pump	Groß-Kienitz, 15831 Blankenfelde-Mahlow
Surface water	08.06.2021	From Teltowkanal (canal), filtrated through a 0.45-μm regenerated cellulose filter	Ernst-Ruska-Ufer, 12489 Berlin

### Immunoassay procedure

All assays were carried out in transparent 96-well flat bottom non-binding polystyrene microplates from Corning (Corning, NY, USA). Wells were filled using Eppendorf Research® pro multichannel pipettes. Dilutions were prepared with Eppendorf Research® plus piston stroke pipettes. Incubation was performed on a Titramax 101 orbital shaker from Heidolph Instruments (Schwabach, Germany). For washing, a BioMag® 96-Well Plate Separator from Polysciences was used. Absorbance measurements were carried out on a SpectraMax® Plus 384 or SpectraMax® i3x microplate reader from Molecular Devices (San José, CA, USA).

For one 96-well plate, 150 µL of DCF-coupled beads suspension was mixed with 4.8 mL of assay buffer. A volume of 50 µL of the resulting suspension was added to each cavity of the microplate. To this, 100 µL/well of DCF standard solution or sample was added. The HRP-labeled mouse anti-DCF antibody F01G21 was diluted in assay buffer to a concentration of 37.2 µg/L, and 50 µL of this solution was added to each well. Then, the resulting mixture was incubated at RT and 900 rpm for 20 min.

To remove unbound antibody, washing was performed by placing the microplate on a magnetic separator. After waiting for 120 s for the particles to separate from the suspension, the supernatant solution was carefully removed by gentle pipetting and replaced by 200 µL of washing buffer. Subsequently, the plate was shaken for 30 s at 1050 rpm for complete resuspension of the particles and the previous steps were repeated twice.

Still on the magnetic separator, 200 µL/well of freshly prepared substrate solution (22 mL citrate buffer, 8.5 µL hydrogen peroxide solution (30%, Sigma-Aldrich) and 550 µL TMB stock solution) were added. After the addition was completed, the plate was shaken for 15 min at RT and 900 rpm (1050 rpm for the first 30 s for resuspension of the beads). Blue color developed in wells with low concentration of DCF.

Color development was stopped by placing the plate on the magnetic separator and adding 100 µL/well of 1 M sulfuric acid (J.T.Baker) immediately. Color change from blue to yellow was observed. The plate was further shaken at RT and 750 rpm for 30 s, while particles remained separated on one side of the respective well.

Detection was performed by reading the optical density at RT at a wavelength of 450 nm with reference at 620 nm. For calibration, O.D. values were plotted against the concentration of standard solutions and a four-parameter logistic function was fitted to the data points. Concentrations of samples (24 per plate, each analyzed in triplicate) were determined by correlating O.D. values to the respective concentration of DCF standards (8 per plate in triplicate).

### LC–MS/MS analysis

LC–MS/MS measurements were carried out on an Agilent 1260 LC system from Agilent Technologies (Waldbronn, Germany) equipped with a binary pump (G1312B), column oven (G1316A), autosampler (G1367E), and a diode array detector (G1315D) coupled to a Triple Quad 6500 Mass Spectrometer from AB Sciex Instruments (Darmstadt, Germany). A Kinetex® 2.6 µm XB-C18 100 Å LC column (150 × 3 mm) from Phenomenex (Aschaffenburg, Germany) and a matching pre-column were used.

Each water sample was analyzed undiluted in duplicate with an injection volume of 10 µL and the column oven temperature set to 55 °C. At a flow rate of 350 µL/min, a binary gradient consisting of (A) water and (B) methanol (LC–MS grade, Biosolve, Valkenswaard, Netherlands) both containing 10 mM ammonium acetate (Sigma-Aldrich) and 0.1% (v/v) acetic acid (Fluka, Buchs, Switzerland) was used under the following conditions: 70% A isocratic for 3 min; linear decrease to 5% A within 9 min; kept at 5% A for 6 min; increase to 70% A within 0.5 min; kept at 70% A for 6 min.

Electrospray ionization (ESI) was performed in positive mode at a source temperature of 400 °C and an ionspray voltage of 4500 V. Gas pressures were applied as follows: curtain gas 35 psi, nebulizer gas 62 psi, turbo gas 62 psi, collision gas 8 psi. At an entrance potential of 10 V, a declustering potential of 90 V, a cell exit potential of 15 V, and a dwell time of 100 ms for each transition, the mass transitions m/z 296 → 250 and m/z 296 → 214 with a collision energy of 22 V (m/z 296 → 250), and 30 V (m/z 296 → 214) were used for quantification in selected reaction monitoring mode. For calibration, DCF standard solutions of eight different concentrations in the range from 0.2 to 100 µg/L including one blank were used.

Data acquisition and analysis were performed using the software Analyst 1.7.1 and Sciex OS- Q 1.4.1.20719 from AB Sciex.

## Results and discussion

### Preparation of DCF-coupled beads

Superparamagnetic amino-functionalized microparticles (BioMag® Plus) were coupled with DCF after activation of its carboxyl function via reaction with DCC and NHS. The DCF active ester readily reacts with amino functions on the particle surface, anchoring the DCF moieties via amide bonds. In a first binding test however, the so-prepared beads showed a very high background signal that could be ascribed to non-specific binding (NSB) of the antibody to excess free amino groups on the bead surface. The same was observed for the untreated particles.

In order to reduce NSB, amino functions were blocked by reaction with cyclic anhydrides, namely succinic anhydride (SA) or glutaric anhydride (GA). Binding tests with two different monoclonal anti-DCF antibodies (F01G21 and SK60-2E4) revealed that NSB was successfully blocked and binding of the antibodies to the DCF moieties was not impaired (Fig. S1).

Interestingly, the preparation of the beads had to be performed in a one-pot reaction with both DCF active ester and anhydride added at once (Fig. S2). Sequential reaction with DCF active ester and anhydride yielded beads that gave high NSB indicating that only the component added first is reacting. Accordingly, addition of anhydride before DCF active ester led to formation of beads which showed no binding of anti-DCF antibodies.

Besides that, incorporation of the C_6_ spacer 6-aminohexanoic acid (Ahx) between DCF and the bead surface (S0 and Fig. S3) was tested as well but appeared to be unsuitable as binding of the anti-DCF antibodies was too strong and could only be inhibited by higher DCF concentrations for the antibody F01G21 (Fig. S4) or not inhibited by up to 10 mg/L DCF for SK60-2E4. On the other hand, the antibody SK60-2E4 showed consistently higher IC_50_ values for DCF beads (Fig. S5) showing the lower affinity of this antibody. Moreover, SK60-2E4 showed binding to beads that were coupled with Boc-protected Ahx which could be inhibited by high DCF concentrations (Fig. S6) indicating lower specificity of that antibody. Against this backdrop, the following investigations were carried out with the antibody F01G21 only.

**Fig. 2:**
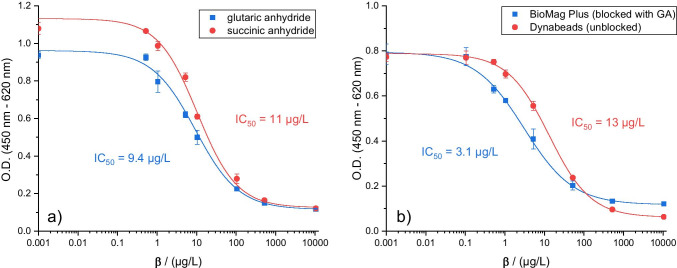
Calibration curves obtained with differently prepared beads (*n* = 3, error bars represent single standard deviation). **a** Comparison of the two blocking reagents GA and SA applied together with DCF active ester on BioMag Plus® particles. **b** Influence of blocking comparing BioMag Plus® particles blocked with GA after further optimization and unblocked Dynabeads™

In a next step, the ratio of DCF active ester and anhydride added to the bead suspension was optimized to achieve the lowest limit of detection (LOD) in binding of dissolved DCF (from samples). It was found that lower amounts of DCF active ester lead to more reproducible results regarding maximum O.D. values after binding of the antibodies. Furthermore, a large excess of anhydride (50–100-fold) over DCF active ester yielded calibration curves with the lowest IC_50_ values at reasonable binding intensities. Comparing both anhydrides, beads that were blocked with GA yielded slightly lower IC_50_ values in calibration curves than those blocked with SA (Fig. 2a). Moreover, the advantage of using a blocking reagent as additional parameter for tuning the binding properties of the antibody could be demonstrated by coupling DCF to a different brand of beads (Dynabeads™ M-270 Amine). These did not require additional blocking as they did not show any NSB of the antibody. In fact, applying a blocking reagent together with DCF active ester prevented binding of anti-DCF antibodies to the beads completely. The unblocked beads, however, allowed binding but calibration curves obtained with these beads showed a significantly higher IC_50_ value than BioMag® Plus beads blocked with GA (Fig. 2b).

Finally, the optimized coupling ratios for reproducible preparation of the DCF-coupled beads were determined to be 0.5 µmol DCF active ester and 50 µmol GA per 100 µL of bead suspension. Beads produced in this manner were used to set up the competitive immunoassay for determination of DCF in water samples.

### Building the competitive immunoassay

Before setting up and optimizing the assay with the antibody F01G21, the recognition element was labeled with horseradish peroxidase (HRP) using a commercial labeling kit. With an achieved coupling ratio of 2.65, the sensitivity of the assay could be increased drastically with lower amounts of antibody needed to obtain high signal intensities. Besides, the assay could be sped up as the additional incubation with a secondary antibody as well as the associated washing step could be omitted.

Assay conditions were optimized regarding the assay buffer, incubation time, amount of bead suspension, and antibody dilution. Regarding the assay buffer, it was found that a Tris-based buffer with EDTA at pH 7.6 was most suitable. While a change in buffer composition and pH did not lead to evident shifts of the calibration curve in terms of the IC_50_, the ratios of minimum and maximum O.D. varied distinctly with the broadest range observed for Tris–EDTA at pH 7.6 (Fig. S7a). Buffers PBS (pH 7.6) and Tris–EDTA (pH 8.5) gave the lowest background signal but also yielded very low maximum signal intensities, requiring more beads and/or antibody to be increased. Both would make the assay less cost-effective. A lower pH of 6.5 with PBS as a buffer did not seem reasonable as here the background signal was the highest of all tested buffers with the signal maximum well below that of Tris–EDTA (pH 7.6). For the purpose of analyzing water samples, the use of Tris–EDTA (pH 7.6) appeared further useful since the contained EDTA can reduce the impact on antibody binding by chaotropic ions like Ca^2+^ and Mg^2+^ which may be contained in higher concentrations in the samples.

The incubation time of the antibody with the sample and beads was found to be sufficient to reach a desired maximum O.D. of 1 ± 0.1 reproducibly after 20 min (Fig. S7b). Longer incubation times not only further increased the maximum intensity but also led to higher background signals from NSB. Therefore, and in the interest of short analysis times, incubation was stopped after 20 min, and beads were washed to remove unbound antibody.

**Fig. 3:**
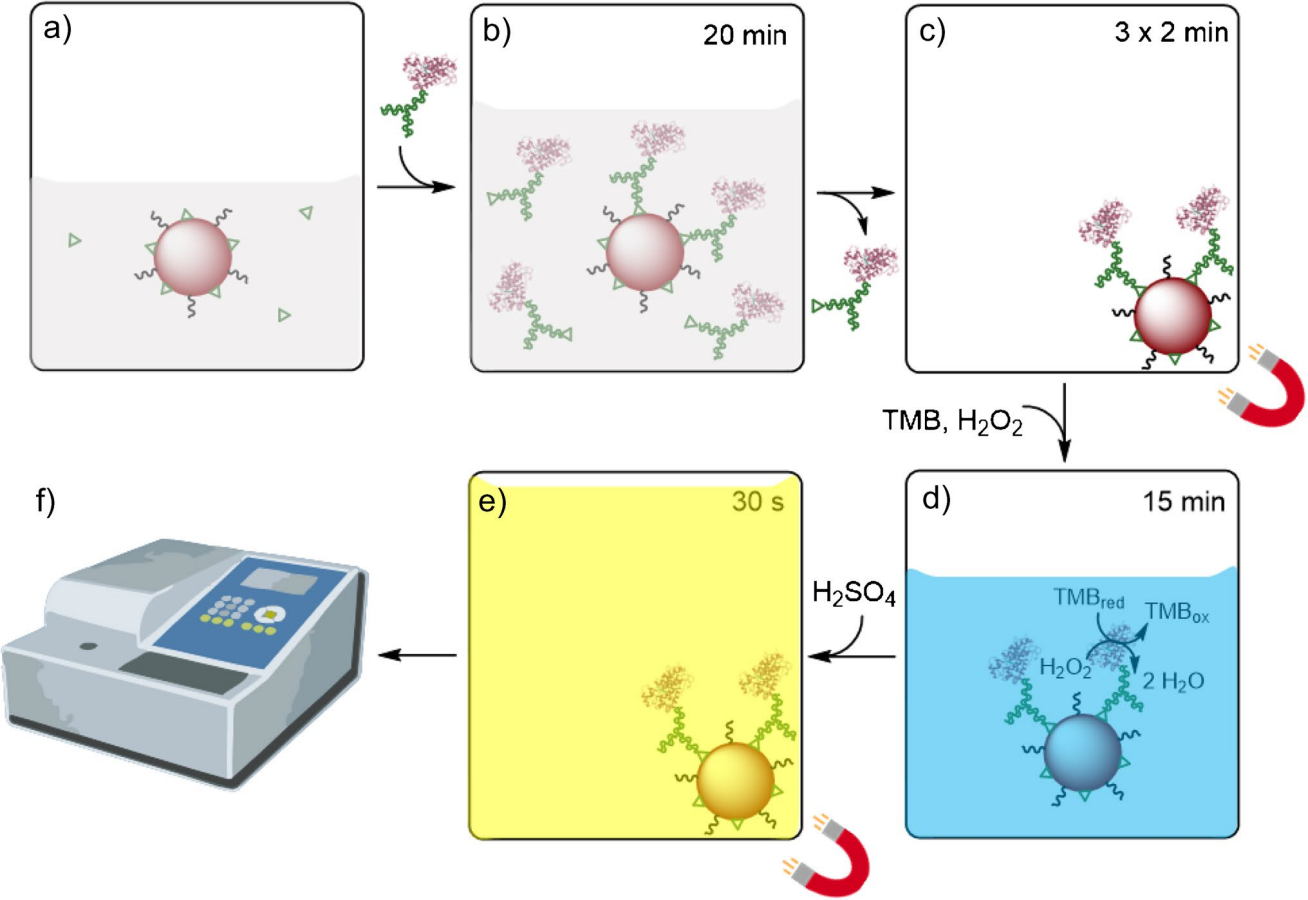
Schematic illustration of each assay step and the respective time frame. **a** Water sample containing DCF (green triangles) is added to the suspension of DCF-coupled magnetic beads in assay buffer. **b** HRP-labeled anti-DCF antibody is added and incubated with the sample and beads for competitive binding for 20 min. **c** Washing is performed by holding the beads with a magnet, removing the supernatant, and adding washing buffer (repeated twice). **d** Substrate solution containing TMB and hydrogen peroxide is added and incubated with the particles for 15 min while blue color develops upon substrate oxidation. **e** The oxidation reaction is stopped by addition of sulfuric acid with the color of the solution changing to yellow, and beads are immobilized on the side of the well for the following **f** absorption measurement in a spectrophotometer

The antibody itself was diluted to a concentration of 9.3 µg/L which yielded reasonable signal intensities at sufficiently low IC_50_ values with an amount of 150 µL of bead suspension per plate which corresponds to approximately 1.5 µL of bead suspension per well. All steps of the immunoassay procedure are illustrated in Fig. 3.

**Fig. 4:**
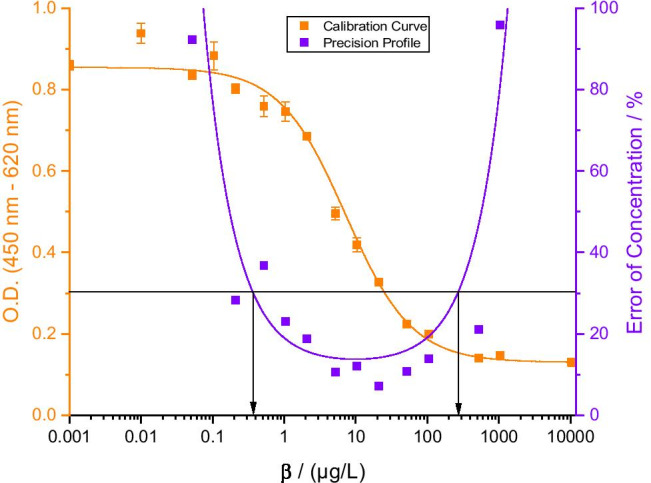
Calibration curve of the optimized assay (orange) and precision profile (purple); *n* = 6; error bars represent single standard deviation. The arrows indicate lower and upper limits of detection, and thus the measurement range of the assay (400 ng/L–300 µg/L)

Under these optimized conditions, the measurement range of the assay was determined according to the rules to set up a precision profile described by Ekins [47] (Fig. 4). Correspondingly, DCF concentrations can be determined with an error of concentration below 30% in a range from 400 ng/L to 300 µg/L. Compared to the previously reported ELISA based on the same antibody [24, 25], this represents a clear improvement (measurement range: 3 – 150 µg/L). With the LOD significantly reduced and the analytical range broadened, the newly developed assay is more versatile and can be used not only for wastewater analysis but also for surface and drinking water analysis as shown below. For the latter, the German Environment Agency (UBA) suggests limit values for DCF of 1.75 µg/L (guidance value) and 20 µg/L (technical action value) [48]. Both these values lie well within the range of this assay. Therefore, it could serve as a quick method to estimate the safety of drinking water. Moreover, with a time of analysis of around 45 min accompanied by the high sample throughput, the magnetic bead-based assay provides analytical results much faster than HPLC–MS and conventional immunoassays like ELISA.

### Analysis of water samples

In order to demonstrate the practicability of the assay for analyzing the concentration of diclofenac in water, six different water samples were taken and analyzed directly as well as at three different spiking levels (24 samples in total). The results were confirmed by LC–MS/MS analysis (Table S1).

**Fig. 5:**
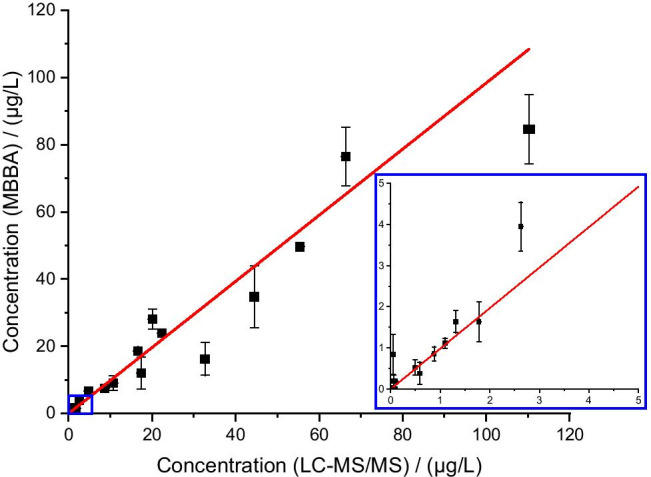
Correlation of DCF concentrations determined by MBBA (*n* = 3) and LC–MS/MS analysis (*n* = 4); error bars represent single standard deviation (regression line parameters: slope *m* = 0.98 ± 0.03, *R*^2^ = 0.976)

Comparison of DCF concentrations determined by MBBA and LC–MS/MS showed good correlation over the covered concentration range from 500 ng/L to 100 µg/L with few larger deviations occurring at elevated concentration values (Fig. 5, Fig. S8, and Table S4). No false negative results were observed demonstrating that contamination of water with DCF could be detected reliably. On the other hand, only one false positive result occurred within the measurement range of the assay (P9) and another one outside of the measurement range (P17) with a determined concentration (203 ng/L) below the actual LOD of the assay. Apart from that, the MBBA was able to detect contamination of the unspiked surface water sample (Teltowkanal) with approx. 500 ng/L DCF which was confirmed by LC–MS/MS. In accordance with this, higher concentrations were found for all three corresponding spiked surface water samples by both methods. Compared with the results of earlier analyses of the same water in 2016 (DCF concentrations: 2.1 and 1.9 µg/L) [21], the concentration determined here appears significantly lower but may be dependent on the sampling site. It is known that an inlet for treated wastewater is further downstream from the sampling site so that higher concentrations of DCF should be found there.

**Fig. 6:**
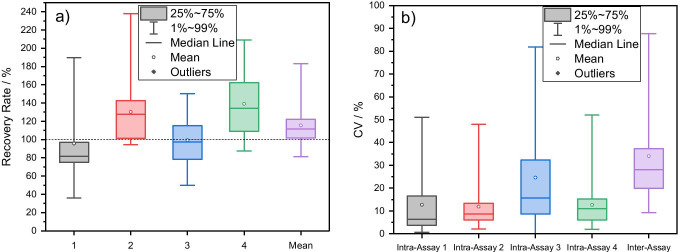
**a** Recovery rates (without blanks, *n* = 19) in four independent analyses and mean of all four measurements for water samples compared to LC–MS/MS reference. **b** Intra-assay variations of four independent sample analyses in triplicate each (*n* = 3, *m* = 24) and inter-assay variation of the mean of these four measurements (*n* = 4, *m* = 24)

In order to evaluate the accuracy of the assay, recovery rates were determined with respect to LC–MS/MS reference measurements. In four different analyses of the same 24 samples, mean recovery rates were found in a range from 96 to 139% with overestimations at low concentration levels shifting the data to higher values (Fig. 6a, Tables S2–S6).

Regarding precision, the mean intra-assay variations of the same four sample analyses ranged from 13 to 25% (Fig. 6b, Tables S2–S6). Higher relative variations were found mainly for low concentration samples. As expected, inter-assay variances were slightly higher with a mean CV of 34% where single false-positive results of blank samples stretched the data range to higher values. Overall, accuracy and precision of the assay appear reasonable for quick estimations of the DCF concentrations in the various tested water samples with the main strengths of the method lying in the high sample throughput, the broad measurement range, short analysis times, cost-effectiveness, and the low equipment expenditure.

## Conclusion

The developed MBBA proved to be a fast and reliable method to determine the DCF concentration in water samples with an LOD of 400 ng/L. Due to the wide measurement range and short analysis time, the MBBA possesses distinct advantages over other immunoassays such as ELISA, which is more time-consuming and in case of DCF less sensitive using the same antibody [24, 25]. This might be the case for other anti-DCF antibodies as well and appears worth investigating [22, 23]. In terms of accuracy and precision, the assay shows satisfactory mean recovery rates of 100–140% in relation to reference analysis by LC–MS/MS, and typical coefficients of variation of 10–25% (intra-assay) and ~ 30% (inter-assay). The MBBA only requires manual washing steps, using a pipette, and therefore does not require a microplate washer which makes its implementation easier and enhances its field portability. With this, the assay provides a quick and easy method to assess contamination of water and in this context the safety of its use as a source of drinking water.

Prospectively, the assay principle can be transferred to other analytes with the same improvement in terms of analysis time and sensitivity as for DCF, having the potential to replace ELISA as the standard technique in immunoanalysis. Incorporation of the magnetic beads into a lateral flow system appears feasible as well and represents another future application of our magnetic beads. As shown previously, changing the detection mode from optical to electrochemical detection is also possible which allows for further miniaturization of the system and mobile testing [49]. Beyond that, the use of magnetic beads will also enable automation and implementation of the assay into autonomous sensors for possible on-site analysis. Our efforts in this direction focus on developing an integrated diagnosis system for pharmaceutical contaminants directly in water supply pipes.

## Supplementary Information

Below is the link to the electronic supplementary material.

Supplementary file1 (PDF 313 KB)

## Data Availability

Supplementary Material is available…
