# Supplementary material for: A rapid magnetic bead-based immunoassay for sensitive determination of diclofenac
Source: Anal Bioanal Chem. 2021 Nov 20;414(4):1563–73. doi: 10.1007/s00216-021-03778-7 (PMC8761716; doi:10.1007/s00216-021-03778-7)
Supplement: Supplementary file 1 — Supplementary file1 (PDF 313 KB) [file 216_2021_3778_MOESM1_ESM.pdf]

A Rapid Magnetic Bead-based Immunoassay for Sensitive Determination of Diclofenac

Supplementary Material

Alexander Ecke[1,2], Tanja Westphalen[1], Jane Hornung[3], Michael Voetz[3], Rudolf J. Schneider[1,4,*]

1 Bundesanstalt für Materialforschung und -prüfung (BAM), Department of Analytical Chemistry; Reference Materials, 12489 Berlin, Germany

2 Humboldt-Universität zu Berlin, Department of Chemistry, 12489 Berlin, Germany

3 sifin diagnostics gmbh, 13088 Berlin, Germany

4 Technische Universität Berlin, Faculty III Process Sciences, 10623 Berlin, Germany

* Corresponding author:

Dr. Rudolf J. Schneider

Bundesanstalt für Materialforschung und -prüfung (BAM)

Department of Analytical Chemistry; Reference Materials

Richard-Willstätter-Str. 11, 12489 Berlin, Germany

rudolf.schneider@bam.de

S0 Preparation of Boc-Ahx Beads & DCF-Ahx Beads

NHS-activation of Boc-Ahx. Analogously to DCF activation, a solution of Boc-Ahx (Sigma-Aldrich) in dry DMF (1/6 mol/L) was prepared under argon. Stock solutions of NHS and DCC of 1/2 mol/L each in DMF were prepared under argon. To the Boc-Ahx solution, NHS solution (1.2 eq), a spatula tip of DSC, and DCC solution (1.2 eq) were added in this particular order under argon. The resulting solution was shaken in the dark at RT and 750 rpm for 18 h. Afterwards, the mixture was centrifuged at 4 °C and 4000 rpm for 10 min in order to separate the precipitated dicyclohexylurea. The supernatant was used directly for coupling.

Coupling to magnetic beads. A suspension of amino-functionalized magnetic microparticles (100 µL) in absolute ethanol (500 µL) and 0.1 M sodium bicarbonate (500 µL) was prepared. Consecutively, 250 µmol of glutaric anhydride or succinic anhydride (Sigma-Aldrich) and 25 µL of the above described Boc-Ahx active ester solution were added. The resulting mixture was shaken at RT and 900 rpm for 20 h. Thereafter, beads were washed once with Milli-Q water (1 mL) and thrice with absolute ethanol (1 mL) using a magnetic separator to hold the beads while removing the supernatant. The Boc-Ahx beads were then resuspended in absolute ethanol (1 mL) and stored at 4 °C until further use.

Deprotection of Boc-Ahx-Beads. Beads were separated from the suspension by applying a magnetic separator and the solvent was removed. Beads were resuspended in 1 mL trifluoroacetic acid (Sigma-Aldrich) and the resulting mixture was shaken for 30 min at RT and 750 rpm. Afterwards, beads were washed once with 1 M sodium bicarbonate, once with Milli-Q water, and twice with absolute ethanol (1 mL each). Beads were then resuspended in absolute ethanol (1 mL) and used directly for coupling with DCF.

Coupling with NHS-activated DCF. To the suspension of deprotected Ahx beads, 25 µL of DCF NHS ester solution (for preparation refer to main article) was added, and the resulting mixture shaken at RT and 900 rpm for 18 h. Afterwards, DCF-Ahx beads were washed once with Milli-Q water and thrice with absolute ethanol (1 mL each), resuspended in absolute ethanol (1 ml) and stored at 4 °C until further use.

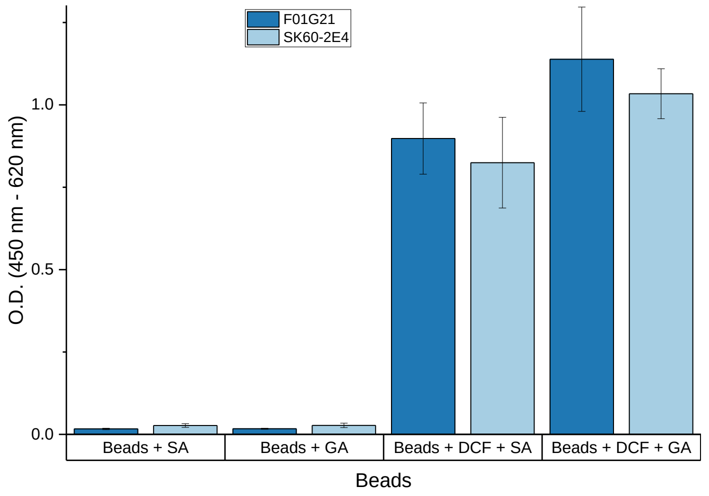

Fig. S1 Optical density values obtained with differently prepared beads after subsequent incubation with monoclonal mouse anti-DCF antibody (F01G21 or SK60-2E4) and secondary antibody (sheep anti-mouse IgG with peroxidase label). Enzymatically converted substrate (TMB) yields colored solution which indicates binding of the antibodies

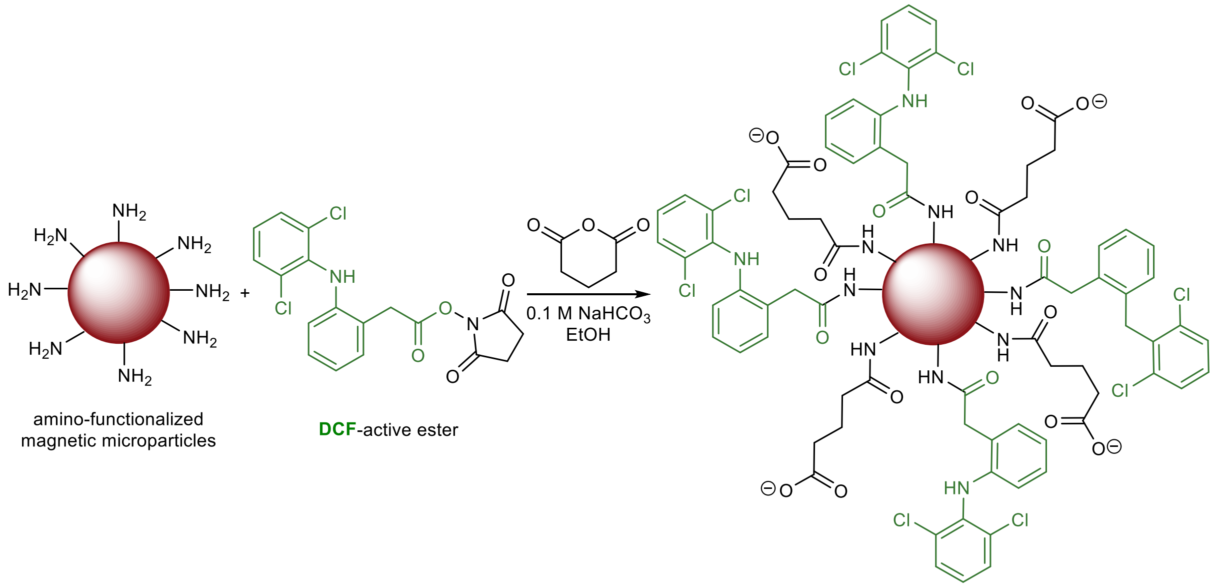

Fig. S2 Reaction scheme for preparation of DCF-coupled beads in a one-pot reaction of amino-functionalized magnetic microparticles with DCF active ester and glutaric anhydride

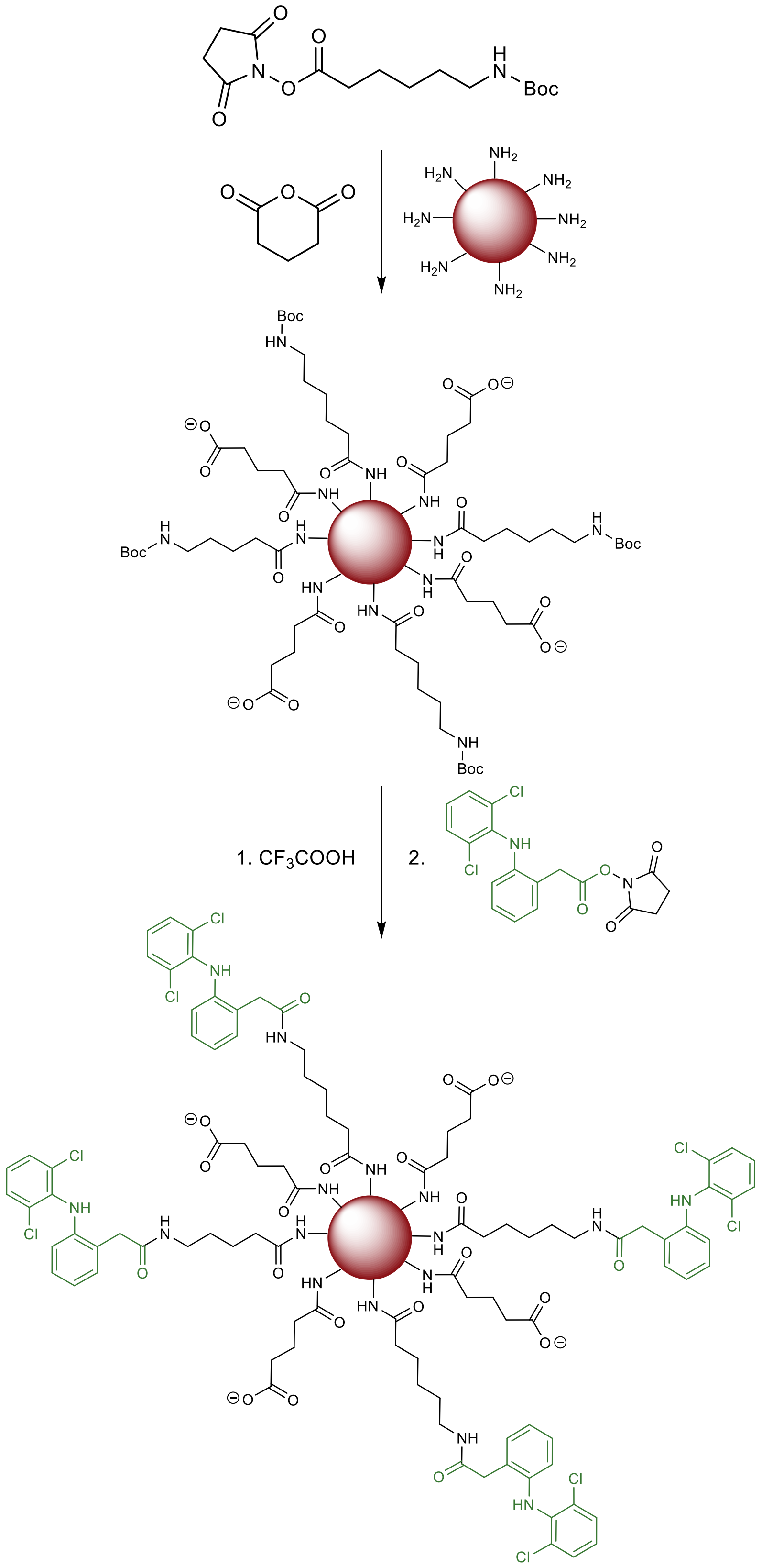

Fig. S3 Reaction sequence for preparation of Boc-Ahx beads and DCF-Ahx beads blocked with glutaric anhydride

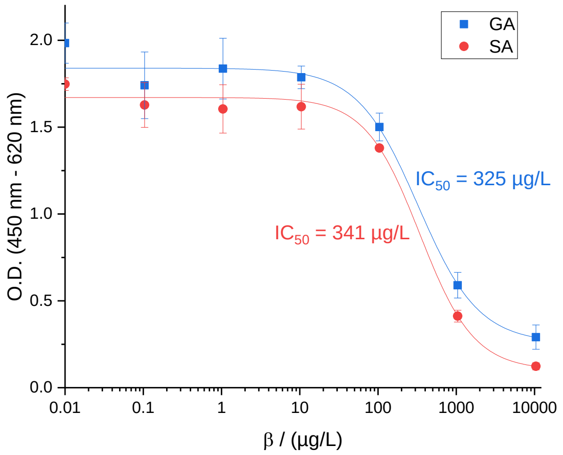

Fig. S4 MBBA calibration curves obtained with DCF-Ahx beads (blocked with glutaric anhydride or succinic anhydride) and anti-DCF antibody F01G21

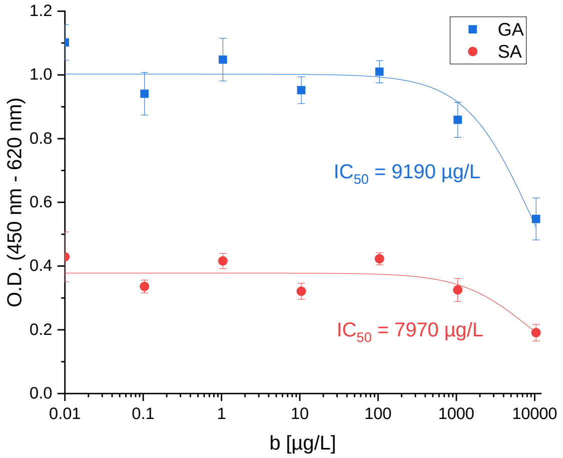

Fig. S5 MBBA calibration curves obtained with DCF beads (blocked with glutaric anhydride or succinic anhydride) and anti-DCF antibody SK60-2E4

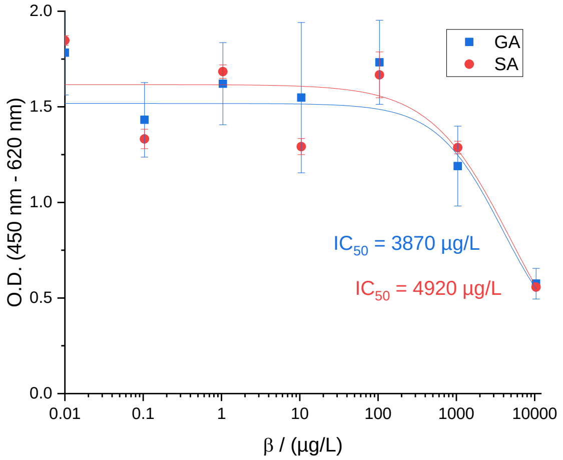

Fig. S6 MBBA calibration curves obtained with Boc-Ahx beads (blocked with glutaric anhydride or succinic anhydride) and anti-DCF antibody SK60-2E4

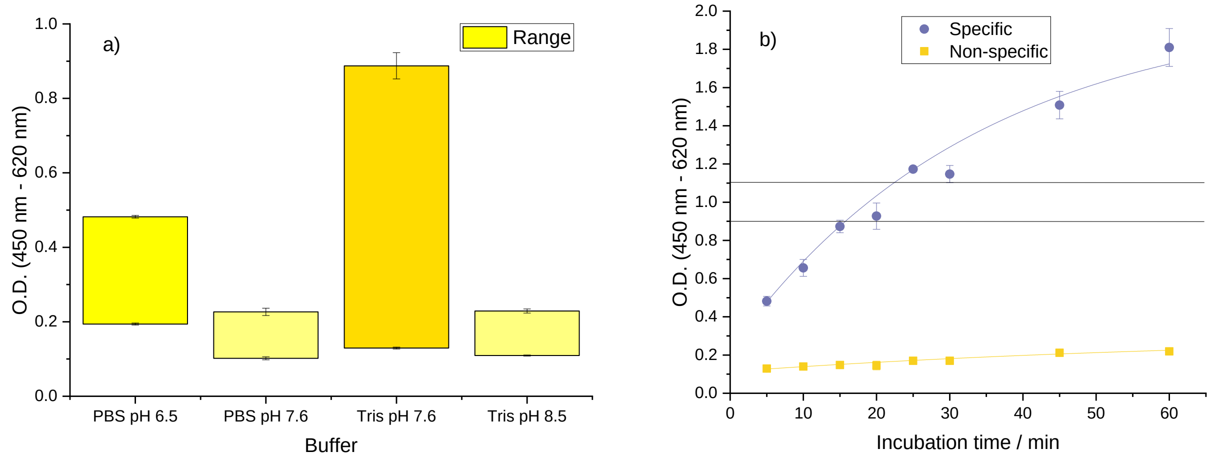

Fig. S7 Influence of a) different assay buffers on the signal range, and b) different incubation times with the antibody on signal intensity

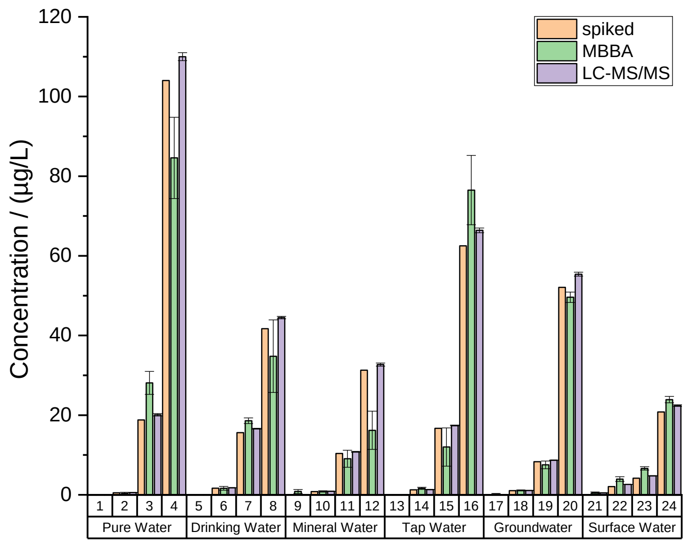

Fig. S8 Comparison of spiked DCF concentrations with the values determined by MBBA and LC-MS/MS

Table S1 Data for LC-MS/MS analysis of water samples (MT = mass transition, Dev. = standard deviation)

Sample	MT 1, value 1 / (µg/L)	MT 1, value 2	MT 2, value 1	MT 2, value 2	Mean	Dev.	CV / %	Spike value / (µg/L)	Δ	Δ_{rel} / %
W1	0.06	0.10	0.00	0.00	0.04	0.04	106	0	0.04	n.a.
W2	0.59	0.60	0.59	0.58	0.59	0.01	1	0.52	0.07	13
W3	19.9	20.1	19.8	20.5	20.1	0.3	1	18.8	1.3	7
W4	109	111	109	112	110	1	1	104	6.3	6
W5	0.10	0.14	0.00	0.05	0.07	0.05	73	0	0.07	n.a.
W6	1.76	1.80	1.76	1.80	1.78	0.02	1	1.67	0.11	7
W7	16.5	16.6	16.5	16.8	16.6	0.1	1	15.6	1.00	6
W8	44.2	44.4	44.3	44.9	44.5	0.3	1	41.7	2.75	7
W9	0.07	0.11	0.00	0.00	0.05	0.05	105	0	0.05	n.a.
W10	0.90	0.92	0.82	0.87	0.88	0.04	4	0.83	0.05	6
W11	10.7	10.8	10.7	10.9	10.8	0.1	1	10.4	0.38	4
W12	32.2	32.7	32.5	33.2	32.7	0.4	1	31.3	1.35	4
W13	0.09	0.12	0.00	0.00	0.05	0.05	102	0	0.05	n.a.
W14	1.37	1.32	1.28	1.29	1.32	0.04	3	1.25	0.07	5
W15	17.2	17.4	17.3	17.5	17.4	0.1	1	16.7	0.65	4
W16	65.9	65.6	66.7	67.2	66.4	0.6	1	62.5	3.85	6
W17	0.12	0.15	0.00	0.00	0.07	0.07	101	0	0.07	n.a.
W18	1.16	1.06	1.08	1.09	1.10	0.04	3	1.04	0.06	6
W19	8.68	8.66	8.70	8.70	8.69	0.02	0	8.33	0.35	4
W20	55.2	54.7	55.6	56.1	55.4	0.5	1	52.1	3.3	6
W21	0.52	0.54	0.45	0.46	0.49	0.04	8	0	0.49	n.a.
W22	2.59	2.65	2.60	2.66	2.63	0.03	1	2.08	0.55	26
W23	4.73	4.74	4.73	4.85	4.76	0.05	1	4.17	0.59	14
W24	22.1	22.2	22.4	22.7	22.4	0.2	1	20.8	1.6	7

Table S2 Data for MBBA measurement 1 (RR = recovery rate)

Sample	Values	Mean Value / (µg/L)	Dev.	CV / %	Spike value	Reference value / (µg/L)	RR / %
W1	1.90<br>1.89<br>1.75	1.85	0.07	4	0	0.04	false positive
W2	1.08<br>0.91<br>1.36	1.12	0.19	17	0.52	0.59	189
W3	14.4<br>19.2<br>13.9	15.8	2.4	15	18.8	20.1	79
W4	94.1<br>69.5<br>95.9	86.5	12.0	14	104	110	79
W5	0.31<br>0.27<br>0.43	0.34	0.07	20	0	0.07	okay
W6	1.14<br>1.51<br>1.04	1.23	0.20	16	1.67	1.78	69
W7	15.7<br>15.7<br>15.9	15.8	0.1	1	15.6	16.6	95
W8	43.3<br>41.8<br>44.3	43.1	1.0	2	41.7	44.4	97
W9	0.41<br>0.59<br>0.30	0.43	0.12	28	0	0.05	okay
W10	1.70<br>1.75<br>1.53	1.66	0.09	6	0.83	0.88	189
W11	10.8<br>9.76<br>11.0	10.5	0.5	5	10.4	10.8	97
W12	26.7<br>27.7<br>24.9	26.4	1.2	4	31.3	32.7	81
W13	0.51<br>0.47<br>0.46	0.48	0.02	5	0	0.05	okay
W14	2.13<br>2.21<br>2.34	2.23	0.09	4	1.25	1.32	169
W15	15.8<br>16.5<br>13.2	15.2	1.4	9	16.7	17.4	87
W16	65.5<br>54.6<br>63.6	61.2	4.8	8	62.5	66.4	92
W17	0.15<br>0.03<br>0.13	0.10	0.05	51	0	0.07	okay
W18	0.72<br>1.02<br>0.48	0.74	0.22	30	1.04	1.10	67
W19	7.49<br>6.96<br>6.96	7.14	0.25	4	8.33	8.68	82
W20	40.9<br>41.8<br>41.8	41.5	0.4	1	52.1	55.4	75
W21	0.18<br>0.28<br>0.07	0.18	0.09	49	0	0.49	36
W22	2.35<br>2.00<br>2.09	2.15	0.15	7	2.08	2.63	82
W23	3.83<br>3.61<br>3.52	3.65	0.13	4	4.17	4.76	77
W24	16.6<br>16.7<br>15.9	16.4	0.4	2	20.8	22.3	74

Table S3 Data for MBBA measurement 2

Sample	Values	Mean Value / (µg/L)	Dev.	CV / %	Spike value	Reference value / (µg/L)	RR / %
W1	1.48<br>1.69<br>1.59	1.59	0.09	5	0	0.04	false positive
W2	0.93<br>0.88<br>0.68	0.83	0.11	13	0.52	0.59	141
W3	18.7<br>18.4<br>19.6	18.9	0.5	3	18.8	20.1	94
W4	139<br>178<br>178	165	18	11	104	110	150
W5	0.41<br>0.41<br>0.44	0.42	0.01	3	0	0.07	okay
W6	3.03<br>2.72<br>3.32	3.02	0.24	8	1.67	1.78	170
W7	17.3<br>16.7<br>16.4	16.8	0.4	2	15.6	16.6	101
W8	62.1<br>57.9<br>50.1	56.7	5.0	9	41.7	44.4	128
W9	1.17<br>1.11<br>1.85	1.38	0.34	24	0	0.05	false positive
W10	1.98<br>1.96<br>2.35	2.10	0.18	9	0.83	0.88	238
W11	15.6<br>14.5<br>15.1	15.1	0.4	3	10.4	10.8	140
W12	49.3<br>37.7<br>52.5	46.5	6.4	14	31.3	32.7	142
W13	0.69<br>0.50<br>0.77	0.65	0.11	17	0	0.05	false positive
W14	1.75<br>1.60<br>1.99	1.78	0.16	9	1.25	1.32	135
W15	--<br>17.0<br>18.1	17.6	0.6	3	16.7	17.4	101
W16	57.9<br>64.4<br>68.2	63.5	4.3	7	62.5	66.4	96
W17	0.72<br>1.67<br>0.59	0.99	0.48	48	0	0.07	false positive
W18	1.19<br>1.69<br>0.92	1.27	0.32	25	1.04	1.10	115
W19	9.78<br>12.1<br>9.23	10.4	1.2	12	8.33	8.68	119
W20	63.3<br>--<br>54.2	58.8	4.6	8	52.1	55.4	106
W21	0.71<br>1.09<br>0.57	0.79	0.22	28	0	0.49	161
W22	3.39<br>4.04<br>3.23	3.55	0.35	10	2.08	2.63	135
W23	4.66<br>4.98<br>4.18	4.61	0.33	7	4.17	4.76	97
W24	23.3<br>25.0<br>20.9	23.1	1.7	7	20.8	22.3	103

Table S4 Data for MBBA measurement 3, visualized in Figure 5 and Figure S8

Sample	Values	Mean Value / (µg/L)	Dev.	CV / %	Spike value	Reference value / (µg/L)	RR / %
W1	0<br>0<br>0	0	0	0	0	0.04	okay
W2	0.05<br>0.38<br>0.72	0.38	0.27	71	0.52	0.59	65
W3	31.5<br>28.4<br>24.3	28.1	2.9	11	18.8	20.1	140
W4	76.8<br>99.1<br>78.0	84.6	10.2	12	104	110	77
W5	0<br>0<br>0	0	0	0	0	0.07	okay
W6	2.24<br>1.60<br>1.07	1.64	0.48	29	1.67	1.78	92
W7	18.8<br>19.4<br>17.7	18.6	0.7	4	15.6	16.6	112
W8	--<br>25.6<br>43.9	34.8	9.1	26	41.7	44.4	78
W9	1.43<br>0.86<br>0.24	0.84	0.49	58	0	0.05	false positive
W10	0.74<br>1.10<br>0.72	0.85	0.17	20	0.83	0.88	97
W11	6.18<br>11.3<br>9.74	9.07	2.14	24	10.4	10.8	84
W12	23.0<br>11.9<br>13.8	16.2	4.8	30	31.3	32.7	50
W13	0.04<br>0.00<br>0.08	0.04	0.03	82	0	0.05	okay
W14	1.54<br>1.37<br>2.00	1.64	0.27	16	1.25	1.32	124
W15	6.15<br>12.1<br>17.8	12.0	4.8	40	16.7	17.4	69
W16	66.3<br>87.5<br>75.6	76.5	8.7	11	62.5	66.4	115
W17	0.39<br>0.06<br>0.15	0.20	0.14	70	0	0.07	okay
W18	1.02<br>1.03<br>1.29	1.11	0.12	11	1.04	1.10	101
W19	7.10<br>8.87<br>6.59	7.52	0.98	13	8.33	8.68	87
W20	49.0<br>51.4<br>48.5	49.6	1.3	3	52.1	55.4	90
W21	0.68<br>0.63<br>0.27	0.53	0.18	35	0	0.49	107
W22	3.75<br>4.75<br>3.34	3.95	0.59	15	2.08	2.63	150
W23	6.18<br>6.52<br>7.18	6.63	0.42	6	4.17	4.76	139
W24	24.5<br>22.7<br>24.5	23.9	0.8	4	20.8	22.3	107

Table S5 Data for MBBA measurement 4

Sample	Values / (µg/L)	Mean Value / (µg/L)	Dev. / (µg/L)	CV / %	Spike value / (µg/L)	Reference value / (µg/L)	RR / %
W1	0.23<br>0.41<br>0.10	0.25	0.13	52	0	0.04	okay
W2	0.59<br>0.84<br>0.55	0.66	0.13	19	0.52	0.59	112
W3	22.0<br>24.8<br>21.0	22.6	1.6	7	18.8	20.1	112
W4	103<br>138<br>101	114	17	15	104	110	104
W5	1.08<br>--<br>0.88	0.98	0.10	10	0	0.07	false positive
W6	2.54<br>3.44<br>2.51	2.83	0.43	15	1.67	1.78	159
W7	20.6<br>24.8<br>19.0	21.5	2.4	11	15.6	16.6	129
W8	68.4<br>87.0<br>60.8	72.1	11.0	15	41.7	44.4	162
W9	1.32<br>1.24<br>1.36	1.31	0.05	4	0	0.05	false positive
W10	--<br>1.64<br>2.03	1.84	0.19	11	0.83	0.88	209
W11	--<br>15.1<br>16.4	15.8	0.6	4	10.4	10.8	146
W12	31.5<br>38.3<br>33.8	34.5	2.8	8	31.3	32.7	106
W13	1.07<br>0.87<br>1.27	1.07	0.16	15	0	0.05	false positive
W14	2.47<br>2.18<br>2.29	2.31	0.12	5	1.25	1.32	175
W15	18.8<br>18.1<br>20.1	19.0	0.8	4	16.7	17.4	109
W16	116<br>108<br>135	120	11	9	62.5	66.4	180
W17	0.92<br>0.59<br>0.70	0.74	0.14	19	0	0.07	false positive
W18	1.38<br>1.19<br>0.94	1.17	0.18	15	1.04	1.10	106
W19	12.7<br>10.8<br>7.29	10.3	2.2	22	8.33	8.68	118
W20	60.8<br>41.5<br>43.0	48.4	8.8	18	52.1	55.4	87
W21	0.76<br>1.05<br>0.89	0.90	0.12	13	0	0.49	184
W22	4.38<br>4.02<br>4.25	4.22	0.15	4	2.08	2.63	160
W23	6.66<br>6.73<br>5.76	6.38	0.44	7	4.17	4.76	134
W24	33.3<br>33.8<br>32.3	33.1	0.62	2	20.8	22.3	149

Table S6 Data for inter-assay variation and mean recovery rates

Sample	Mean value 1	Mean value 2	Mean value 3 / (µg/L)	Mean value 4	Mean All	Dev.	CV / %	Reference value / (µg/L)	RR / %
W1	1.85	1.58	0.00	0.25	0.92	0.81	88	0.04	false positive
W2	1.11	0.83	0.38	0.66	0.75	0.27	36	0.59	126
W3	15.9	18.9	28.1	22.6	21.4	4.6	21	20.1	106
W4	86.5	165	84.6	114	113	32	29	110	102
W5	0.34	0.42	0.00	0.98	0.44	0.35	81	0.07	okay
W6	1.23	3.02	1.64	2.83	2.18	0.76	35	1.78	122
W7	15.7	16.8	18.6	21.5	18.2	2.2	12	16.6	109
W8	43.1	56.7	34.8	72.1	51.7	14.1	27	44.4	116
W9	0.43	1.38	0.84	1.31	0.99	0.38	39	0.05	false positive
W10	1.66	2.10	0.85	1.83	1.61	0.47	29	0.88	183
W11	10.5	15.1	9.1	15.8	12.6	2.9	23	10.8	117
W12	26.4	46.5	16.2	34.5	30.9	11.1	36	32.7	94
W13	0.48	0.65	0.04	1.07	0.56	0.37	66	0.05	false positive
W14	2.22	1.78	1.64	2.32	1.99	0.29	14	1.32	151
W15	15.2	17.6	12.0	19.0	16.0	2.7	17	17.4	92
W16	61.2	63.5	76.5	120	80.3	23.7	29	66.4	121
W17	0.10	0.99	0.20	0.74	0.51	0.37	73	0.07	false positive
W18	0.74	1.27	1.11	1.17	1.07	0.20	19	1.10	98
W19	7.14	10.4	7.52	10.3	8.84	1.52	17	8.68	102
W20	41.5	40.1	49.6	48.4	44.9	4.2	9	55.4	81
W21	0.17	0.79	0.53	0.90	0.60	0.28	47	0.49	122
W22	2.15	3.55	3.95	4.22	3.47	0.80	23	2.63	132
W23	3.65	4.61	6.63	6.38	5.32	1.24	23	4.76	112
W24	16.4	23.1	23.9	33.1	24.1	5.9	25	22.3	108
